# Effect of Ligament Fibers on Dynamics of Synthetic, Self-Oscillating Vocal Folds in a Biomimetic Larynx Model

**DOI:** 10.3390/bioengineering10101130

**Published:** 2023-09-26

**Authors:** Bogac Tur, Lucia Gühring, Olaf Wendler, Samuel Schlicht, Dietmar Drummer, Stefan Kniesburges

**Affiliations:** 1 Division of Phoniatrics and Pediatric Audiology, Department of Otorhinolaryngology, Head and Neck Surgery, University Hospital Erlangen, Medical School, Friedrich-Alexander-Universität Erlangen-Nürnberg, Waldstrasse 1, 91054 Erlangen, Germany; 2 Institute of Polymer Technology, Friedrich-Alexander-Universität Erlangen-Nürnberg, Am Weichselgarten 10, 91058 Erlangen, Germany

**Keywords:** synthetic vocal fold models, integrated fibers, biomimetic larynx model, physiological vocal fold dynamics

## Abstract

Synthetic silicone larynx models are essential for understanding the biomechanics of physiological and pathological vocal fold vibrations. The aim of this study is to investigate the effects of artificial ligament fibers on vocal fold vibrations in a synthetic larynx model, which is capable of replicating physiological laryngeal functions such as elongation, abduction, and adduction. A multi-layer silicone model with different mechanical properties for the musculus vocalis and the lamina propria consisting of ligament and mucosa was used. Ligament fibers of various diameters and break resistances were cast into the vocal folds and tested at different tension levels. An electromechanical setup was developed to mimic laryngeal physiology. The measurements included high-speed video recordings of vocal fold vibrations, subglottal pressure and acoustic. For the evaluation of the vibration characteristics, all measured values were evaluated and compared with parameters from ex and in vivo studies. The fundamental frequency of the synthetic larynx model was found to be approximately 200–520 Hz depending on integrated fiber types and tension levels. This range of the fundamental frequency corresponds to the reproduction of a female normal and singing voice range. The investigated voice parameters from vocal fold vibration, acoustics, and subglottal pressure were within normal value ranges from ex and in vivo studies. The integration of ligament fibers leads to an increase in the fundamental frequency with increasing airflow, while the tensioning of the ligament fibers remains constant. In addition, a tension increase in the fibers also generates a rise in the fundamental frequency delivering the physiological expectation of the dynamic behavior of vocal folds.

## 1. Introduction

Human phonation is a multifaceted physical process influenced by a myriad of factors. Humans have the remarkable ability to manipulate their larynx to affect their voice. For instance, the fundamental frequency $F_{0}$ can be increased by tensing the vocal folds, a process in which the pre-phonatory vocal fold posturing plays a pivotal role.

The impact of these vocal fold postures is particularly observable in voice pathologies, such as atrophy [1,2,3,4] or paresis [5]. These studies underscore the critical influence of glottal closure on human phonation [6,7,8]. Even in physiological female and child phonation, interestingly, incomplete glottis closure postures occur without pathological voice characteristics [7,9,10].

To investigate the fundamental influence of vocal fold postures on dynamics, ex vivo experiments are highly suitable [11,12,13]. However, these experiments face challenges such as the scarcity of human larynges and their individuality, which makes it sometimes difficult to formulate universally valid statements. Furthermore, from an experimental perspective, these larynges also need to be processed quickly to prevent degeneration.

For these reasons, synthetic vocal fold models made of silicone have been developed to better investigate the human phonation process [14]. These synthetic models offer the advantage of parametrizable geometry and material properties, and they can be reproducibly fabricated. Artificial larynx models are used to measure the vocal folds collision forces [15,16,17,18,19,20,21], characterize supraglottal aerodynamics [22], serve as validation models in developing advanced laryngoscopic techniques [23,24,25], estimate energy transfer in the vocal folds [26,27], investigate acoustic interaction [28], and study the asymmetric behavior of vocal folds [29,30]. Many research papers report an incomplete glottal closure in synthetic, self-oscillating vocal fold models [31,32,33]. Reasons for incomplete glottis closure include the isotropic properties of the silicone used for the vocal folds. In contrast, physiological vocal fold vibrations tend to complete glottal closure under various laryngeal postures [1]. In past studies [32], attempts were made by applying vertical restraint to the lateral half of the superior surface of the synthetic vocal folds. It was reported that this led to complete glottal closure due to increased stiffness in the anterior–posterior direction through elongation of the vocal folds [34]. Hirano and Kakita [35] showed that the integration of a muscle layer as well as collagen and elastin fibers in the lamina propria achieved transverse isotropy. Murray and Thomson [36] reproduced glottis closure with a combination of an epithelium layer and an extremely soft cover. In addition, a fiber was included into the ligament, which reinforced the effect of stiffness in the anterior–posterior direction. To represent the physical properties of the collagen and elastin fibers, the study by Shaw et al. [37] was carried out. They cast acrylic and polyester fibers into the surface layer to achieve non-linear stress–strain characteristics of the vocal folds. Xuan and Zhang [38] focus in their study on achieving glottal closure with an epithelium layer and embedded fiber in a single-layer silicone model.

The few models summarized above focused on specific and restricted phonatoric conditions with regard to pre-phonatoric posturing or fiber tension separately. Therefore, the aim of the study presented here is to introduce a synthetic larynx model that is able to reproduce both, different types of pre-phonatoric posturing (elongation and ad-/abduction) combined with different tension levels of the embedded fibers in the ligament layer of the vocal folds model. In this model, fibers are cast into a multi-layer synthetic vocal fold model based on the M5 geometry by Scherer et al. [39] in the anterior–posterior direction. These fibers are tensioned under mechanical force to investigate the effects on the $F_{0}$ and the subglottal pressure $P_{sub}$ with an initially pre-phonatory closed glottis. Additionally, this study also examines typical voice parameters from high-speed videos and acoustic measurements, to contextualize the results within the physiology of vocal fold dynamics.

## 2. Materials and Methods

### 2.1. Synthetic Larynx Model

**Figure 1:**
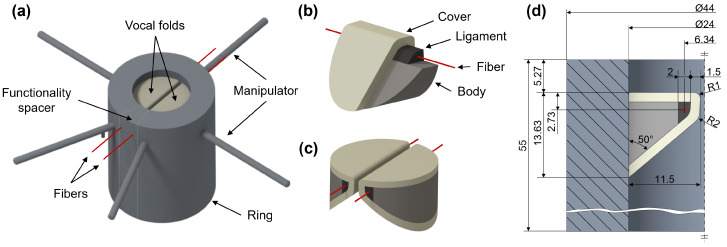
(**a**) 3D CAD model of the entire larynx model, with the manipulators, the vocal folds based on the M5 model, the ring and the fibers (**b**) View of the individual layers in the vocal fold model, with the body, the ligament, the fiber and the cover (**c**) 3D perspective view of the vocal folds as they are arranged in the larynx model (**d**) Mid-coronal cut showing the exact dimensions in mm of the entire model and the position of the fibers.

The synthetic larynx model includes a vocal fold model made of silicone with the M5 geometry by Scherer et al. [39], which is cast into a silicone ring as shown in Figure 1a. This ring incorporates additional manipulators at various positions, designed to mimic the typical laryngeal functions of ad-/abduction and elongation during pre-phonatory posturing of the vocal folds. The silicone vocal folds are structured in different layers capturing the musculus vocalis and the lamina propria, comprising the ligament and mucosa as shown in Figure 1b,c. Characteristic biomechanical properties have been selected for each layer during the fabrication process [36,40]. Furthermore, fibers are integrated into the ligament to mimic the fibrous structure in the human vocal fold ligament. Figure 1d provides detailed geometric parameters in a mid-coronal cut. Two types of platinum-catalyzed two component silicone rubber (Smooth-On, Inc., Macungie, PA, USA) with specific proportions of silicone thinner are employed to fabricate the larynx model: Ecoflex 00-30, Dragon Skin 10 Slow and Silicone Thinner.

The silicone ring embodies a balance between rigidity and flexibility essential for laryngeal functions. For this reason, the ring was made of Dragon Skin 10 Slow. For all other elements of the model, Ecoflex 00-30 was cast with a certain amount of thinner. Table 1 shows the different proportions associated with the constituents of the entire larynx model.

In the present study, a total of six different larynx models (MLM1-MLM6) each with a different fiber type were examined to evaluate the resulting vibratory responses of the vocal folds (see Table 2). The diameters of these fibers ranged from 0.108 mm to 0.3 mm, with corresponding break resistances from 1.18 kg to 7.9 kg. The thinnest of these tested fibers is composed of polyvinylidene fluoride (PVDF), while the remainder are made from polyamide 6.6 (PA6.6). In our study, we determinded the mechanical properties of the storage modulus E${}^{\prime }$, loss modulus E${}^{\prime \prime }$, and the loss tangent tan δ (the ratio of E${}^{\prime \prime }$ and E${}^{\prime }$). The dynamic characterization of the applied pre-conditioned fibers is conducted using a solid analyzer of type RSA-G2 (TA Instruments, New Castle, DE, USA). To compensate for moisture-dependent effects of polyamide filaments, all samples are prepared under normal conditions (23 °C, 50% humidity) for 30 days. Applied characterizations include frequency sweeps, ranging from 0.5 Hz–50 Hz at a constant strain of 0.1%, and amplitude sweeps at a constant frequency of 1 Hz, with oscillation strains ranging from 0.01 to 1%. All measurements are conducted at a controlled temperature of 25 °C.

**Figure 2:**
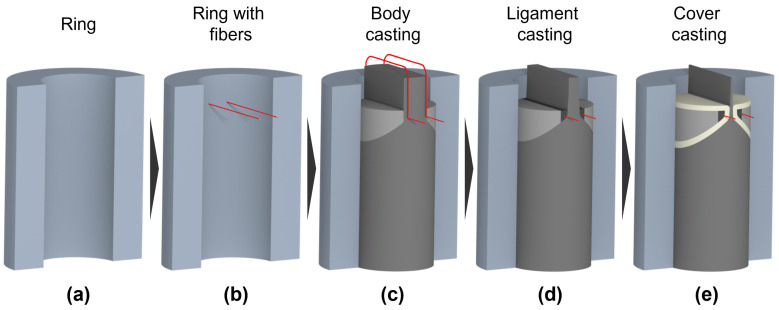
The casting process of the multi-layer vocal folds in the artificial larynx model is shown. (**a**) Ring in the cross-section, (**b**) Ring in the cross-section with integrated fibers, (**c**) Casting of the body, (**d**) Casting of the ligament and (**e**) Casting of the cover.

The casting process of the synthetic larynx model involves several steps, as displayed in Figure 2a,b. Initially, the silicone ring is cast, incorporating a mold for the functionality spacer. Within this process, the five manipulators are embedded within the silicone ring to control the laryngeal functions, as mentioned above. The silicone ring is then demolded and inserted into a mechanical frame that allows for precise positioning of a 0.9 mm thick cannula through the ring, which serves as a guide for threading the fibers. The fibers are then secured, and the cannula is removed, resulting in the fibers being accurately positioned within the ring. This process is replicated for both the left and right vocal fold.

Subsequent steps involve casting the various layers of the vocal folds using different molds: Initially, the body is cast, followed by the ligament, and finally, the cover. Figure 2c–e shows the molding process for the multi-layer vocal fold model. All parts of the model are cured for at least eight hours at a temperature of 40 °C and cooled down to room temperature. To avoid air deposits, the pre-cast liquid silicone mixtures have been degassed under vacuum for at least 10 min before each casting step. At the end of the final step, the surfaces of the vocal folds are dusted with talcum powder to prevent them from adhering to each other after the mold is completely removed. The molds and the manipulators are made of Grey Resin (Formlabs, Somerville, MA, USA) printed with the Formlabs Form 2 stereolithography 3D printer (Formlabs, Somerville, MA, USA).

### 2.2. Biomimetic Functionality

The biomimetic functionality of the synthetic laryngeal model is realized by including an electromechanical control system to reproduce typical laryngeal dynamics such as elongation, adduction, and abduction. This system is facilitated by manipulators that transmit the linear and rotational motion from the electromotors to the silicone ring displaying the laryngeal cartilages (thyroid and arytenoid) and therefore to the vocal folds.

**Figure 3:**
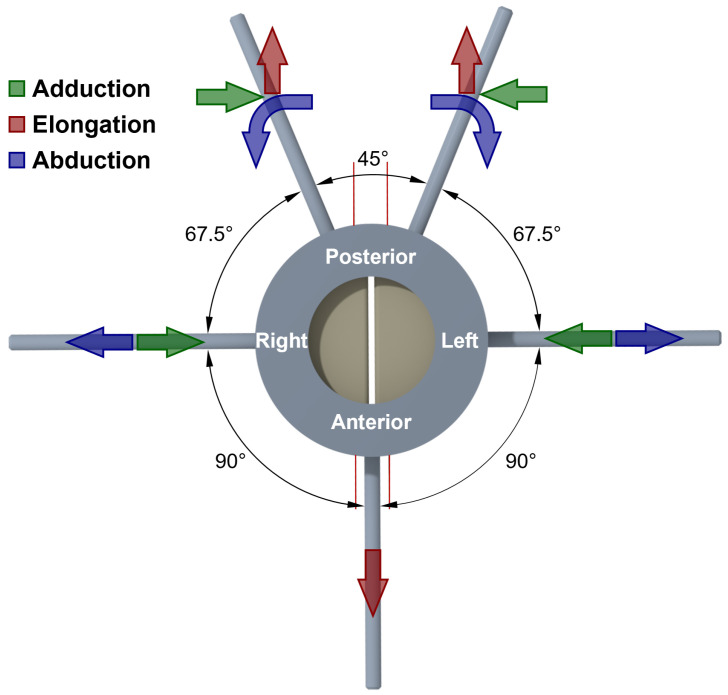
The top view of the artificial larynx model and the manipulators with the angular arrangement. In addition, the laryngeal dynamical motions, i.e., elongation and ad-/abduction, are indicated by colored arrows.

The apparatus incorporates a total of seven motors, each serving a distinct function that perform the linear and rotational motion as displayed in Figure 3. Three of these 8MT173 Motorized Translation Stages (Standa Ltd., Vilnius, Lithuania) are responsible for linear movement to elongate the vocal folds indicated by the red arrows. Two 8MR151 Motorized Rotation Stages (Standa Ltd., Vilnius, Lithuania) are designated for rotational movement to realize the ad-/abduction motion supported by the remaining two Motorzied XY Scanning Stages (Standa Ltd., Vilnius, Lithuania). The ad-/abduction motion is thereby shown with blue and green arrows in Figure 3.

The control of these motorized stages is achieved through an 8SMC5-USB Stepper & DC Motor Controller (Standa Ltd., Vilnius, Lithuania). The motors are controlled by Python scripts which are integrated within a globally working LabVIEW (National Instruments, Austin, TX, USA) measurement script.

### 2.3. Fiber Guidance and Tensioning System

Another laryngeal function integrated in this model is the application of a pretension in the ligament layer of the vocal folds. This is realized by the inclusion of elastic fibers as described above. These fibers are embedded within the ligament layer in an unloaded state. To control the free fiber ends, the fibers are guided through a customized guidance management system designed to minimize frictional loss and to ensure parallel alignment. This is realized by bearing pulleys to reduce friction at redirection units for the fibers.

The fibers are secured to a 7T67-25 Stable Steel Translation Stage (Standa Ltd., Vilnius, Lithuania) capable of linear movement, with a travel range of 25 mm. This setup allows for precise control over the tension of the ligament. By the integration of highly stiff fibers, a non-linear tension can be introduced by the elongation of the vocal folds and the fibers qualitatively similar to human ligament tissue [42,43,44].

### 2.4. Measurement Setup and Data Acquisition

The investigation employed a multimodal measurement setup acquiring different physical parameters from the model. The setup used in this study is a modified version of the setup introduced by Birk [45] for ex vivo larynx models [46,47,48]. The synthetic larynx model was mounted on an artificial trachea with a diameter of 24 mm. Airflow, which induces the oscillations of the vocal folds, was regulated in standard liter per minute (SLM) by a 1579 A/B mass flow controller (MKS, Andover, MA, USA) and a 4000B digital power supply (MKS, Andover, MA, USA). The subglottal pressure signal was measured using an XCS-93-5PSISG pressure sensor (Kulite Semiconductor Products, Inc., Leonia, NJ, USA) connected to a PXIe-4330 bridge module (National Instruments, Austin, TX, USA), with a sampling frequency of 44.1 kHz for 1 s. The pressure sensor is located approx. 130 mm below the glottal level.

The glottal region and the dynamics of vocal fold vibration were examined using a Phantom V2511 digital high-speed camera (Vision Research, Wayne, NJ, USA). The frame rate was set to 4000 frames/s (fps) with a picture resolution of 768 × 768 pixels. A Canon EF 180 mm f/3.5L macro lens (Canon, Inc., Tokyo, Japan) was mounted on the camera to display the vocal folds on the camera chip. The recording duration amounted 600 ms. The start of the high-speed recording was triggered by a PXIe-6356 multifunctional module (National Instruments, Austin, TX, USA).

In the supraglottal region, acoustic signals were sampled with two 4189 1/2-inch free-field microphones (Brüel & Kjær, Nærum, Denmark) at a distance of at least 30 cm from the model, applying a sampling frequency of 44.1 kHz for 1 s duration. Care was taken that the microphones were not exposed to the airflow coming from the model. Both microphones were connected to a Nexus 2690 microphone conditioning amplifier (Brüel & Kjær, Nærum, Denmark). The analog voltage signals were sampled and A/D converted by a PXIe-4492 sound and vibration module (National Instruments, Austin, TX, USA.

The pressure (aerodynamic and acoustic) signals were synchronously sampled using a LabVIEW script. All measurements (microphone, pressure sensor and camera) were simultaneously started.

### 2.5. Data Processing and Analysis

The computation of parameters as $F_{0}$ and mean $P_{sub}$ was performed with MATLAB R2021b (The MathWorks, Inc., Natick, MA, USA). The spectral analysis is based on Welch’s method [49,50], employing a hamming window with a window length of 0.37 s, to yield the power spectral density of the pressure signals [51]. Thereby, the post-processing of the subglottal pressure was conducted relative to the atmospheric pressure.

Glottal dynamic parameters were derived from the high-speed imaging videos of the vocal fold oscillations using our in-house software package Glottis Analysis Tool 2020 (GAT) [52,53]. This tool facilitates the segmentation of the glottis area between the vocal folds to obtain the glottal area waveform (GAW), from which characteristic parameters describing the vocal fold dynamics during phonation are calculated. The chosen parameters for glottal dynamics describe the dynamical behavior regarding the glottal closure, i.e., Glottis gap index (GGI) and Closing quotient (ClQ), the vibration periodicity, i.e., Amplitude periodicity (AP) and Time periodicity (TP), and symmetry of the vocal folds, i.e., Phase asymmetry index (PAI) and Amplitude symmetry index (ASI). For the calculation of these parameters, 20 consecutive cycles were used, which is the minimum number of cycles that provide stable parameters in high-speed video recordings [54,55]. An expanded discourse and relevant literature concerning these parameters can be found in Table 3a.

GAT was also used for the physiological parameter evaluation of acoustic and subglottal pressure. Regularity parameters, Jitter (jitt) and Shimmer (shim), and sound quality parameters, Harmonics-to-noise ratio (HNR), Normalized noise energy (NNE) and Cepstral peak prominence (CPP), were considered. For these analyses, 100 cycles were used [56,57]. Table 3b furnishes additional data and bibliographic references pertinent to these parameters [58].

**Table 3:** Overview of computed (**a**) glottal dynamic parameters and (**b**) acoustic parameters.

Parameter	Abbreviation/Unit	Description/Range	Reference
**(a) Glottal dynamic parameters**			
Glottis gap index	GGI/AU	0: full closure/[0, 1]	[59]
Closing quotient	ClQ/AU	1: completely open/[0, 1]	[60]
Amplitude periodicity	AP/AU	1: periodic/[0, 1]	[61]
Time periodicity	TP/AU	1: periodic/[0, 1]	[61]
Phase asymmetry index	PAI/AU	0: symmetric/[0, 1]	[61]
Amplitude symmetry index	ASI/AU	1: symmetric/[0, 1]	[62]
**(b) Acoustic parameters**			
Harmonics-to-noise ratio	HNR/dB	Higher is better *	[63]
Normalized noise energy	NNE/dB	Smaller is better *	[64]
Cepstral peak prominance	CPP/dB	Higher is better *	[65]
Shimmer	shim/%	Smaller is better *	[66]
Jitter	jitt/%	Smaller is better *	[66]

* The aphorism “Smaller/Higher is better” pertains to the realm of healthy modal phonation, and is to be construed in the context of efficiency, regularity, harmonic richness, and noise levels.

### 2.6. Measuring Protocol

In the course of our investigation, we fabricated six distinct synthetic larynx models, each representing a different fiber type as previously specified in Table 2. The procedure for data collection and measurement was equal for all models. Each model was placed on the artificial trachea, with the manipulators mounted to the motors. Subsequently, the fibers were secured to the linear stage via the fiber guidance and tensioning system. The translation stage, with a travel range of 0–25 mm, was initially set at 5 mm representing the initial tension level in just tight-free condition.

We followed a systematic protocol to collect data from the synthetic larynx models. The process, which was repeated for each model, consisted of the following steps:

1. Relax the translation stage to 0 mm to ensure the fibers are in a completely tension-free state with no force applied.
2. Adduct the vocal folds until complete glottal closure is achieved.
3. Take a reference measurement of the subglottal pressure with the flow completely switched off.
4. Manually increase the flow rate until the oscillation onset flow is identified, which is the point at which the synthetic vocal folds begin to oscillate stably.
5. Record the first measurement at the onset of oscillation.
6. Iteratively increase the flow rate by increments of 10 SLM until reaching the maximum flow rate of 200 SLM.
7. Switch off the flow.
8. Increase the fiber tension by elongating the fiber by 5 mm.
9. Establish complete glottis closure if not already closed.
10. Repeat the procedure starting from identifying the onset flow (step 4).

This procedure was repeated, with each measurement starting from the onset flow after adjusting the fiber tension and ensuring complete glottis closure. Thereby, the maximum elongation of the fiber was 25 mm, which is the maximum tension level. Using this procedure, each larynx model was tested at six tension levels, for each at least 2 flow rate levels.

## 3. Results and Discussion

### 3.1. General Phonation Parameters

**Figure 4:**
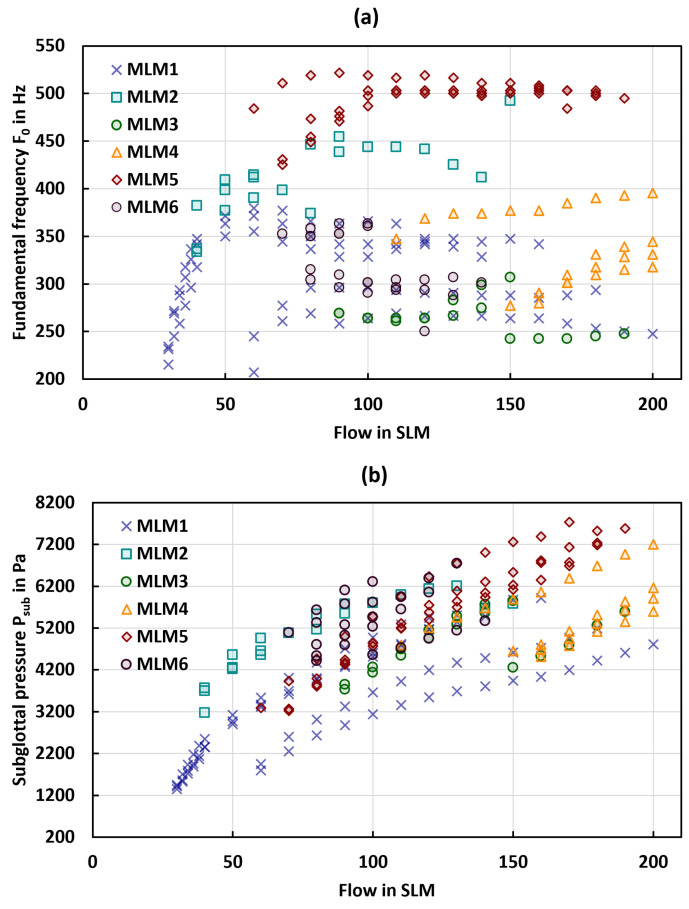
The figure presents the measured parameters (**a**) the $F_{0}$ in Hz and (**b**) the $P_{sub}$ in Pa as a function of flow in SLM. for different tension levels of the fiber.

Within the study, we performed a total of N=213 measurements with six larynx models. The results are presented in Figure 4, with Figure 4a depicting the $F_{0}$ in Hz and Figure 4b illustrating the $P_{sub}$ in Pa as a function of flow in SLM for all models and all tension levels of the fibers. Table 4 provides a comprehensive summary of the general phonation parameters for all models, as well as the parameters at onset.

A noteworthy observation from the data is the wide range of $F_{0}$ values produced by models MLM1 and MLM2, as detailed in Table 4a. In contrast, MLM3–MLM6 show a much smaller $F_{0}$ range than MLM1 and MLM2 with the highest frequencies for MLM5.

The mechanical properties of the individual layers of the synthetic multi-layer vocal fold models, as shown in Table 1, are within physiological ranges of related tissues [43,67,68,69,70,71,72,73]. These properties contribute to the generation of $F_{0}$ values during vocal fold oscillation, which align with the physiological range of human phonation [42,74]. While $F_{0}$ for males typically lies between 100 and 220 Hz [75], females show a slightly higher $F_{0}$ range [76]. The synthetic models presented in this study also demonstrate higher $F_{0}$ values beyond normative human phonation, up to the ranges of professional female singers [77]. In comparison to other models, i.e., ex vivo porcine and ovine [45,47,48] studies as well as other synthetic models [16,18,20,36,38,40], our synthetic model vibrates at significantly higher $F_{0}$ values. Thus, this model enables us to reliably study the phonation process at these high frequencies in the range of professional female singers as mentioned above.

According to Figure 4b, all models exhibited an increase in $P_{sub}$ with increasing flow. Furthermore, a trend was observed indicating that $P_{sub}$ increases with the diameter of the fibers. While MLM1 displayed the largest range in $P_{sub}$, the other models demonstrated higher overall $P_{sub}$ values.

Measuring the $P_{sub}$ in vivo presents significant challenges, leading to a scarcity of publications that can be used to reference the physiological range of the mean $P_{sub}$. Despite this, Holmberg et al. [60] report a $P_{sub}$ for males and females between 580–680 Pa and 600–700 Pa, respectively. In contrast, Sundberg et al. [75,78] report $P_{sub}$ values between 800–2200 Pa, whereas Baken and Orlikoff [79] estimate the $P_{sub}$ values to range from 353–1941 Pa. In our study, the measured $P_{sub}$ values of the synthetic models were predominantly higher than these reported physiological ranges. MLM1, however, lied in these ranges, which will be discussed in more detail in Section 3.5. These higher values of the $P_{sub}$ in synthetic models matched well with many previous studies [31,51,80]. When compared to ex vivo measurements [45,46,47,48,81], the $P_{sub}$ in our models is also higher. However, the $P_{sub}$ increase with increasing flow is similarly reproduced [56,82]. The reason for a higher $P_{sub}$ in the presented models could be the flow resistance by the integrated fibers. While the onset flow rates shown in Table 4 observed in the presented model do not fall within the physiological range typical of normal phonation, it is consistent with other studies that applied synthetic as well as ex vivo larynx models [36,38,83].

### 3.2. Glottal Dynamic Parameters

**Figure 5:**
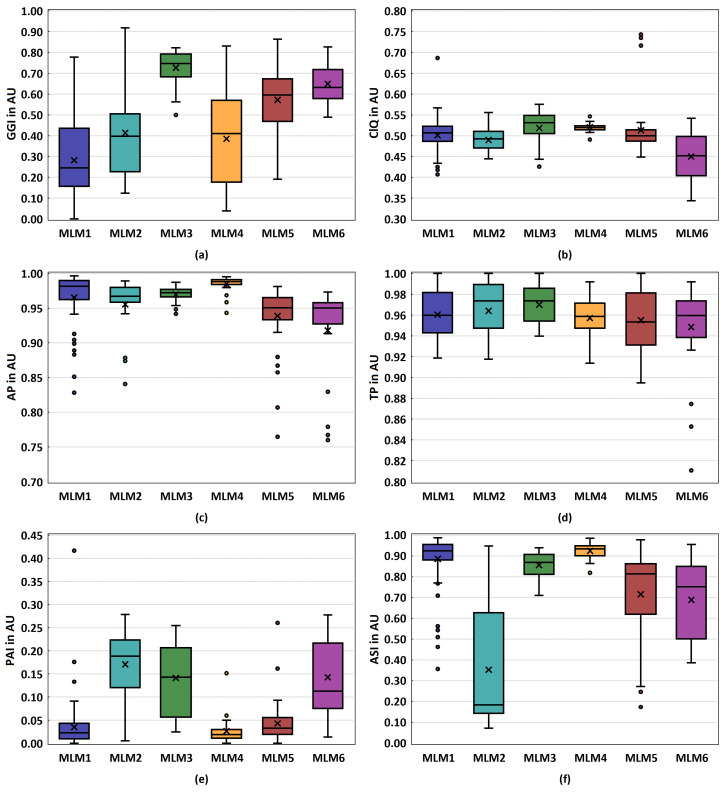
Illustrates boxplots for the glottal dynamics parameters of the models MLM1-MLM6: (**a**) Glottis gap index (GGI) (**b**) Closing quotient (ClQ) (**c**) Amplitude periodicity (AP) (**d**) Time periodicity (TP) (**e**) Phase asymmetry index (PAI) (**f**) Amplitude symmetry index (ASI).

The glottal dynamic parameters are presented as boxplots in Figure 5, with a focus on the glottal gap (GGI, ClQ), periodicity (AP, TP), and symmetry (ASI, PAI) of the vocal folds oscillations.

**Glottal gap parameters:** Except for MLM3 and MLM6, all models exhibit a high variability in the GGI, with MLM3 and MLM4 showing notably high GGI values compared to the rest, see Figure 5a. An observable trend is the increase in the median GGI with increasing fiber diameter, with MLM3 displaying the highest median as an exception. The GGI describes the ratio between the minimum and maximum glottal area and can take values between 0 and 1 (see Table 3a). A GGI value close to 0 indicates large vibrational changes as well as complete glottal closure, whereas values approaching 1 describe less change in the glottal area [59,84].

The GGI ranges of all fabricated synthetic models measured in this study are notably higher compared to those reported in ex vivo [46,47,48] and in vivo [7,85,86,87,88] studies. However, the GGI for the MLM1 comprises the entire range starting with GGI=0 representing regular phonation up to GGI=0.78 representing a severe glottis closure insufficiency [31,89,90].

In terms of the ClQ, which describes the closing time of the glottis per cycle, all models, except for notable outliers for MLM1 and MLM5, fall within the expected range for physiological vocal fold vibration, as seen in Figure 5b. These ClQ values align well with those reported in ex vivo studies involving porcine [45,48], and ovines [47] vocal folds. Furthermore, in vivo studies suggest that the observed ClQ values are representative of healthy phonation [86].

**Periodicity parameters:**

The AP for all models show a median above 0.95, with relatively low variability across all models, see Figure 5c. However, some outliers can be observed. Similarly, for TP, all models also record a median above 0.95, albeit with higher variability than AP, see Figure 5d. Apart from MLM6, no outliers were measured. The synthetic vocal fold models indicate a high degree of periodicity in the amplitude of vocal fold vibration. This is consistent with the expectation for physiological vocal fold vibration [47,48].

**Symmetry parameters:**

MLM1, MLM4, and MLM5 exhibit a very low PAI with minimal variability, shown in Figure 5e. The remaining models display both a higher PAI and significantly greater variability. The ASI for MLM2 shows both the highest variability and the lowest median. While MLM5 and MLM6 are not as low as MLM1–MLM3, MLM4 shows the highest ASI with minimal variability. Apart from MLM1, the remaining models show little or even no outliers, see Figure 5f. The symmetry of vocal fold vibration is a critical aspect of voice production, and asymmetric characteristics of the vocal fold motion can be indicative of vocal pathologies [91,92]. In terms of the PAI, models MLM1, MLM4, and MLM5 exhibit very low values with minimal variability, suggesting a high degree of symmetry of vocal fold vibration. This is in line with the other reports of ex vivo [47,48,86] and in vivo studies [88]. The remaining models display both a higher PAI and significantly greater variability, indicating less symmetry in their vocal fold vibration. Conversely, the high ASI and minimal variability of MLM4 indicate a high degree of symmetry in the amplitude of vocal fold vibration. These findings underscore the importance of considering both the phase and the amplitude symmetry in the evaluation of synthetic vocal fold models.

### 3.3. Acoustic Parameters

**Figure 6:**
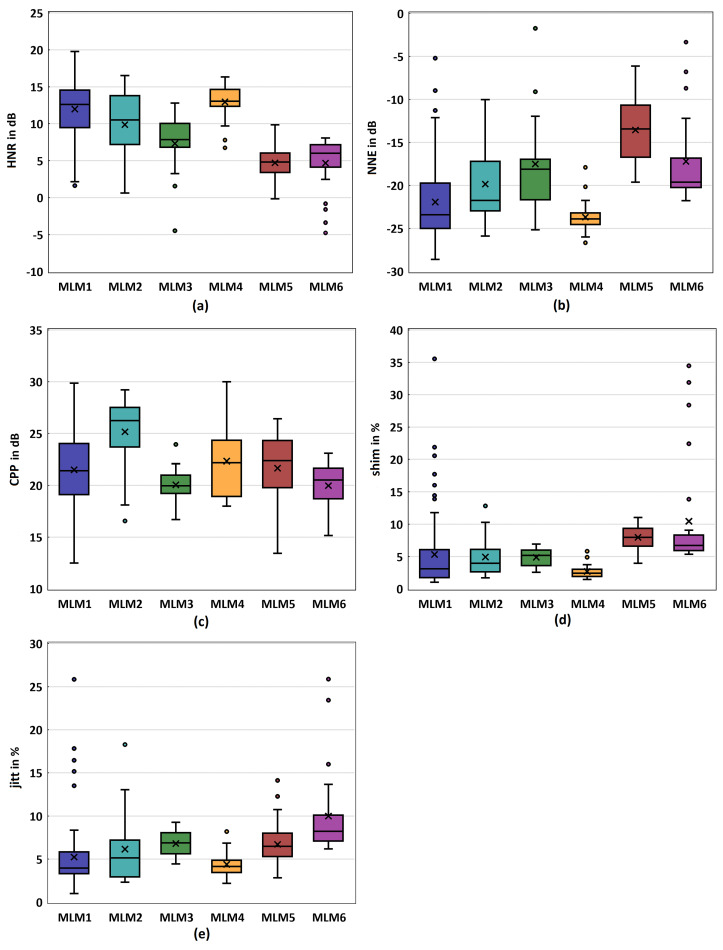
Boxplots for the acoustic parameters of the models MLM1-MLM6: (**a**) Harmonics to noise ratio (HNR) (**b**) Normalized noise energy (NNE) (**c**) Ceptral peak prominance (CCP) (**d**) Shimmer (shim) (**e**) Jitter (jitt).

In this section, we present the acoustic parameters. Interestingly, an observable trend is that the median of the HNR decreases with increasing fiber diameter, with the exception of the MLM4 model. It shows the highest dispersion in the MLM1 model, accompanied by a relatively high median value, see Figure 6a. However, the highest HNR is produced by the MLM4 model, which shows minimal dispersion.

Compared to the literature, the synthetic models exhibit HNR values that are similarly high to those reported in ex vivo studies [46,47,48].

The NNE presents a different pattern. With the exception of the MLM4 and MLM6, an increase in fiber diameter corresponds to an increase in the NNE value, which is shown in Figure 6b. Therein, the MLM1 and MLM4 models exhibited the lowest median values, with MLM1 showing a higher variability. In this context as well, the values fall within a physiological range when compared to ex vivo studies [47].

All models display relatively high CPP values, as shown in Figure 6c, with MLM2 producing the highest median. Figure 6d,e show the computed values for the Jitter and Shimmer parameter. Except for the MLM4 model, all other models demonstrate similar behavior of these two parameters. An observable trend is that the median values increase with the fiber diameter. The lowest values of both Jitter and Shimmer are again shown by the models MLM1 and MLM4, respectively. In comparison to previous studies, the values for CPP, jitt, and shim observed in our synthetic models closely align with those reported in ex vivo experiments [46,47,48].

### 3.4. Evaluation of Reproduction Characteristics of the Larynx Model

Based on the results presented above, the models were evaluated with regard to their ability of reproducing physiological parameters by using a scoring system. This will facilitate the identification of the most suitable model for physiological vocal folds oscillations to illustrate the impact of fiber tension on $F_{0}$ and $P_{sub}$. For each parameter, the six models are rated from the best (score 6) to the worst (score 1) with regard to physiological range. After each model has been scored for each parameter, the total score of each model was calculated by summing up the individual scores of the single parameters for the respective model.

The scoring system takes into account several factors: the number of measurements per model, with more measurements getting a higher score, as shown in Table 4; the range of the fundamental frequency with a higher score for a larger frequency range $F_{0}$ (see Table 3); the glottal flow resistance $R_{B}$ [93] with a larger score for a higher resistance; the glottal dynamic parameters as seen in Table 3a, and the acoustic parameters shown in Table 3b both being closer to their optimum.

The resulting total scores are displayed in Table 5 for each model with MLM1 and MLM4 exhibiting the highest scores, which indicate that the calculated parameters largely fall within the range of physiological vocal fold vibrations. Given that the ranges for the fundamental frequency of both models largely overlap (see Figure 4 and Table 3), the subsequent analysis will focus solely on the MLM1 model with the thinnest fiber. All other results demonstrating the influence of fiber tension are provided in the Supplementary Material.

### 3.5. Influence of Fiber Tension

**Figure 7:**
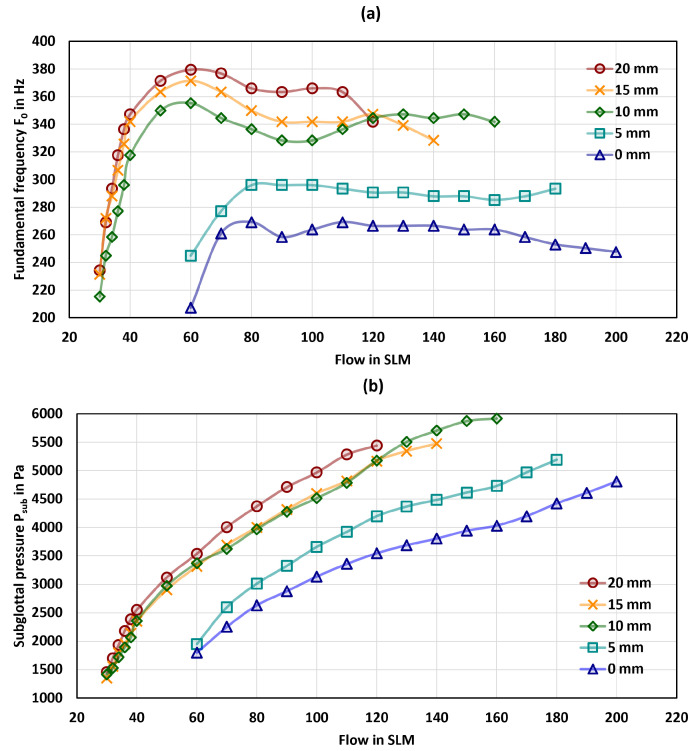
The figure shows the influence of different fiber tension levels for the MLM1 model. Illustrates (**a**) the $F_{0}$ in Hz and (**b**) the $P_{sub}$ in Pa as a function of flow in SLM.

As described in Section 2.3, the fibers in the models were elongated from 0 mm–20 mm in 5 mm increments. Figure 7 shows the effect of the fiber tension on $F_{0}$ and $P_{sub}$ for MLM1. As expected, $F_{0}$ increases with increasing flow for each level of tension. Furthermore, it can be observed that for a flow rate of less than 40 SLM, the frequency increases linearly until it reaches a saturation point at a flow greater than 40 SLM. This effect of the linear increase in $F_{0}$ is particularly noticeable for the tension levels of 10 mm, 15 mm, and 20 mm.

The $P_{sub}$ also exhibits the effect of nearly linear increasing with the flow rate. Similarly, $F_{0}$ and $P_{sub}$ also increase with increasing fiber tension at a constant flow rate. The increase in $F_{0}$ and $P_{sub}$ is highest between the stages of 5 mm and 10 mm. Xuan and Zhang [38] reported an increase in the $F_{0}$ when a fiber was integrated into the body of a synthetic vocal fold model. This model was based on a one-layer isotropic vocal fold model, which served as the baseline for their study [26,32,34]. Interestingly, when the fiber was integrated into the cover of the model, there was no significant change in the $F_{0}$. However, the $P_{sub}$ exhibited a more intriguing behavior. While the integration of the fiber into the body led to a decrease in $P_{sub}$, it increased when the fiber was embedded in the cover. It should be noted that in their study, the fibers were not subjected to any tension. In another study, Murray and Thomson [36] investigated the influence of fiber tension. They employed a multi-layer model and integrated the fibers into the ligament. By applying tension to the fibers using weights at each end, they achieved an increase in $F_{0}$ of approximately 10%.

The integration of fibers into the present synthetic model produced similar effects as reported in other studies about incorporated fibers in synthetic vocal fold models [36,38]. In contrast to these studies, the fibers can be elongated variably, allowing for the control of frequency at specific flow stages. As previously mentioned, the impact of fiber elongation is physiologically relevant, particularly in the range where the $F_{0}$ increases linearly before reaching a saturation point, which was also reported elsewhere [94].

## 4. Conclusions

This study presents the design, construction, and evaluation of a synthetic larynx model, incorporating various functional aspects of human laryngeal dynamics. The model is fabricated using two types of platinum-catalyzed silicone rubber, with integrated manipulators and fibers, to mimic the biomechanical properties of the human larynx and vocal folds. These models are systematically tested with varying fiber types and their elongation, providing a comprehensive understanding of the impact of these variables on vocal fold vibration.

Our analysis demonstrates that these synthetic larynx models are capable of replicating the key features of physiological vocal fold oscillation, as indicated by a wide range of glottal dynamic and acoustic parameters. Despite some disparities, the models exhibit behavior largely in line with the expected physiological up to pathological ranges. Notably, the fundamental frequency $F_{0}$ and subglottal pressure $P_{sub}$ values generated by the synthetic models fall within the physiological ranges reported in in vivo and ex vivo studies, indicating their potential for reproducing the characteristics of human phonation.

It is found that the larynx model presents a wide range of $F_{0}$ values depending on the different fiber types, indicating the crucial role of fiber characteristics within the ligament to control the vibratory responses of the vocal folds. Furthermore, our results show that an increase in fiber diameter corresponds to an increase in $P_{sub}$, indicating an increase in flow resistance due to the greater stiffness of thicker fibers. Further studies will be dedicated to elucidating the correlations between the fiber characteristics and the observed outcomes.

The models’ ability to replicate the periodicity, symmetry, and glottal gap parameters of vocal fold vibration further corroborates their physiological similarity. However, it is also observed that certain configurations of the synthetic models may not fully replicate typical dynamics.

We also evaluate the influence of fiber tension on the $F_{0}$ and $P_{sub}$, revealing that $F_{0}$ increases linearly with increasing flow until reaching a saturation point. This observation shows the high relevance of including fibers into the ligament in synthetic larynx models to accurately reproduce human vocal fold dynamics.

The development and evaluation of these synthetic larynx models offer a significant contribution to the field of voice research. Furthermore, this is the first synthetic larynx model that provides dynamic phonation characteristics of professional female singers. Additionally, the model is able to analyze the regular as well as irregular phonation by varying the pre-phonatory settings of the vocal folds in an asymmetric manner.

## Figures and Tables

**Table 1:** Material properties of the silicone compounds used in various larynx elements, detailing the specific mixing ratios and corresponding Young’s modulus [41].

Larynx Elements	Silicone	Mixing Ratio Part A:Part B:Thinner	Young’s Modulus in kPa
Ring	Dragon Skin 10 Slow	1:1:0	151
Functionality spacer	Ecoflex 00-30	1:1:2.5	6 *
Body	Ecoflex 00-30	1:1:2	8.2
Ligament	Ecoflex 00-30	1:1:0	60
Cover	Ecoflex 00-30	1:1:4	2.5

* The Young’s modulus was estimated using cubic spline interpolation, leveraging available empirical data points for varying proportions of thinner in the mixture.

**Table 2:** Overview of the various fiber-based models MLM1-MLM6 including their material composition, diameter, break resistance, and associated dynamic mechanical properties E${}^{\prime }$-Modulus, E${}^{\prime \prime }$-Modulus and tan δ, using frequency sweeps from 0.5 Hz–50 Hz at a constant strain of 0.1%.

Model	Fiber					
	Material	Diameter in mm	Break Resistance in kg	E ${}^{\prime }$ -Modulus * in GPa	E ${}^{\prime \prime }$ -Modulus * in GPa	tan δ *
MLM1 **	PVDF	0.108	1.18	2.91	0.08	0.03
				(0.07)	(0.01)	(0.00)
MLM2	PA 6.6	0.12	1.4	2.39	0.16	0.07
				(0.04)	(0.00)	(0.00)
MLM3	PA 6.6	0.125	1.82	-	-	-
MLM4	PA 6.6	0.18	2.5	1.19	0.12	0.10
				(0.03)	(0.00)	(0.00)
MLM5	PA 6.6	0.25	5.3	1.82	0.16	0.09
				(0.38)	(0.03)	(0.00)
MLM6	PA 6.6	0.3	7.9	1.71	0.12	0.07
				(0.10)	(0.01)	(0.00)

* The values in parentheses show the standard deviation. ** MLM is the abbreviation for **M**ulti-**L**ayer-**M**odel.

**Table 4:** Range of phonation parameters grouped for the data at the onset flow rate only and the all data with higher flow rate. The parameter N corresponds to the number of measurements conducted for the respective model. The standard deviation is indicated in parentheses besides the mean value.

	MLM1 (N = 76)	MLM2 (N = 20)	MLM3 (N = 18)	MLM4 (N = 26)	MLM5 (N = 48)	MLM6 (N = 25)
**(a) Phonation onset**						
mean $F_{0}$ in Hz	226.63 (13.55)	371.44 (31.71)	260.19 (12.68)	298.77 (28.42)	453.54 (25.92)	328.82 (22.38)
mean $P_{sub}$ in Pa	1593.66 (235.96)	4375.36 (906.12)	3944.00 (223.64)	4661.83 (100.76)	3444.03 (318.67)	4789.36 (325.45)
mean flow rate in SLM	42 (14.69)	66 (38.78)	110 (28.28)	145 (20.61)	70 (7.07)	82 (9.79)
**(b) All recordings**						
mean $F_{0}$ in Hz	306.42 (43.66)	411.28 (38.46)	264.82 (17.65)	337.18 (36.87)	496.32 (20.55)	318.36 (30.43)
max $F_{0}$ in Hz	379.52	492.57	306.84	395.67	522.18	363.37
min $F_{0}$ in Hz	207.25	333.76	242.24	277.23	425.28	250.32
mean $P_{sub}$ in Pa	3595.90 (1281.65)	5026.83 (863.09)	4872.53 (642.95)	5549.97 (708.14)	5702.86 (1245.18)	5405.57 (614.18)
max $P_{sub}$ in Pa	5918.14	6206.71	5851.08	7200.75	7737.04	6745.46
min $P_{sub}$ in Pa	1350.85	3172.69	3733.03	4515.16	3221.27	4417.77

**Table 5:** Comparative evaluation of six larynx models based on physiological parameter reproduction scores (++: 6, +: 5, o+: 4, o-: 3, -: 2, - -: 1).

	MLM1	MLM2	MLM3	MLM4	MLM5	MLM6
N	++	-	- -	o+	+	o-
$F_{0}$ range	++	+	- -	o+	-	o-
$R_{B}$	o-	++	-	- -	o+	+
GGI	++	o+	- -	+	o-	-
ClQ	++	+	o-	-	o+	- -
AP	o+	o-	+	++	-	- -
TP	o+	+	++	o-	-	- -
PAI	+	- -	o-	++	o+	-
ASI	+	- -	o+	++	o-	-
HNR	+	o+	o-	++	-	- -
NNE	+	o+	o-	++	- -	-
CPP	o+	++	-	+	o-	- -
shim	+	o+	o-	++	- -	-
jitt	++	o+	-	+	o-	- -
Score	70	54	39	65	39	27

## Data Availability

The data presented in this study are not publicly available due to ongoing research in this field.

## Supplementary Materials

The following supporting information can be downloaded at: https://www.mdpi.com/article/10.3390/bioengineering10101130/s1, Figures S1–S5: The figures show the influence of different fiber tension levels for the models MLM2-MLM6. Illustrates (a) the $F_{0}$ in Hz and (b) the $P_{sub}$ in Pa as a function of flow in SLM.
