# Supplementary material for: Effect of Ligament Fibers on Dynamics of Synthetic, Self-Oscillating Vocal Folds in a Biomimetic Larynx Model
Source: Bioengineering (Basel). 2023 Sep 26;10(10):1130. doi: 10.3390/bioengineering10101130 (PMC10604794; doi:10.3390/bioengineering10101130)
Supplement: Supplementary file 1 [file bioengineering-10-01130-s001.zip › Tur_MDPI_2023_supfile_submission.pdf]

Supplementary Materials: Effect of ligament fibers on dynamics of synthetic, self-oscillating vocal folds in a biomimetic larynx model

Bogac Tur [1], Lucia Gühring [1], Olaf Wendler [1], Samuel Schlicht [2], Dietmar Drummer [2] and Stefan Kniesburges [1]

1. Influence of fiber tension

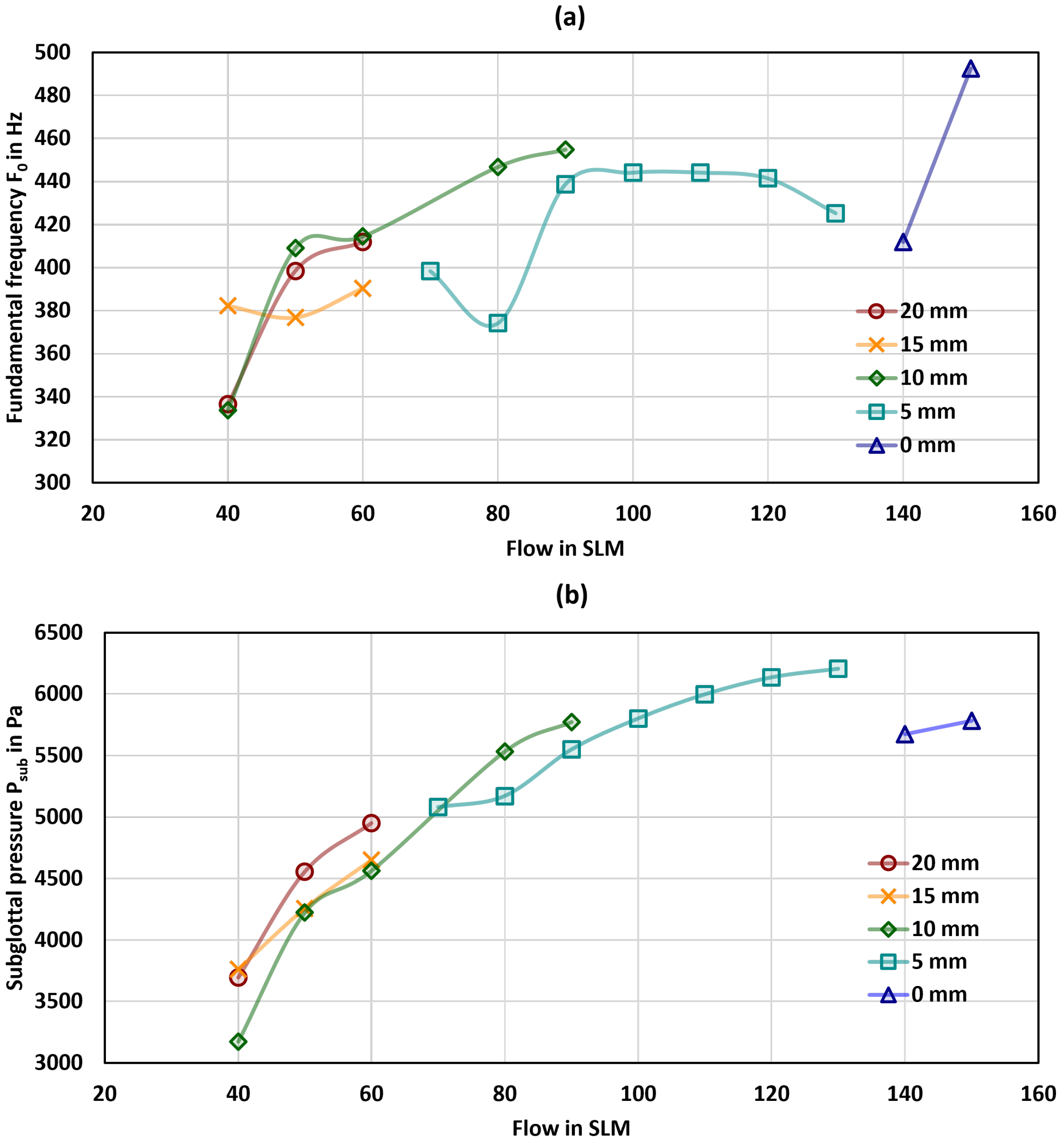

Figure S1. The figure shows the influence of different fiber tension levels for the MLM2 model. Illustrates (**a**) the F_0 in Hz and (**b**) the P_{sub} in Pa as a function of flow in SLM.

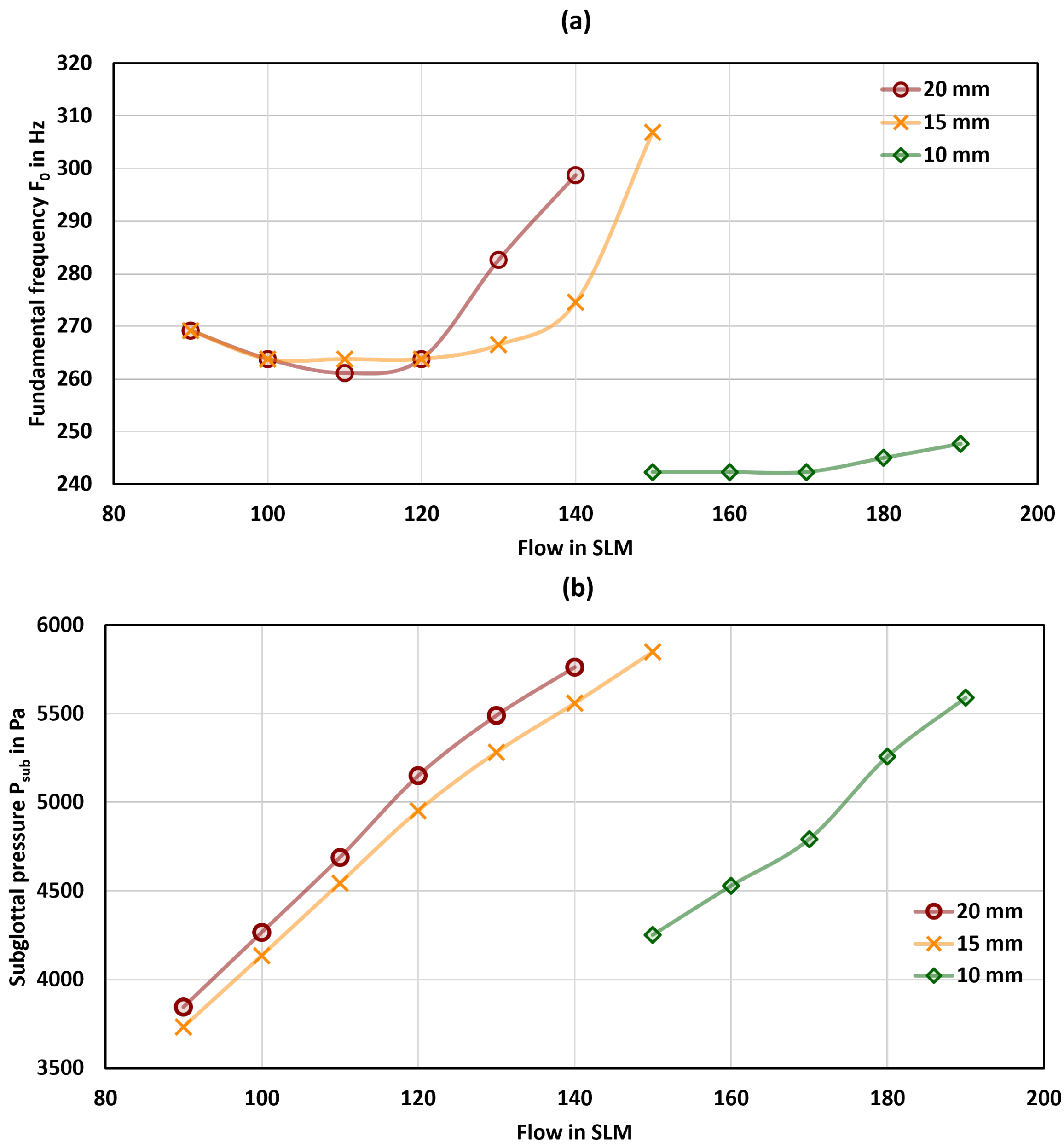

Figure S2. The figure shows the influence of different fiber tension levels for the MLM3 model. Illustrates (**a**) the F_0 in Hz and (**b**) the P_{sub} in Pa as a function of flow in SLM.

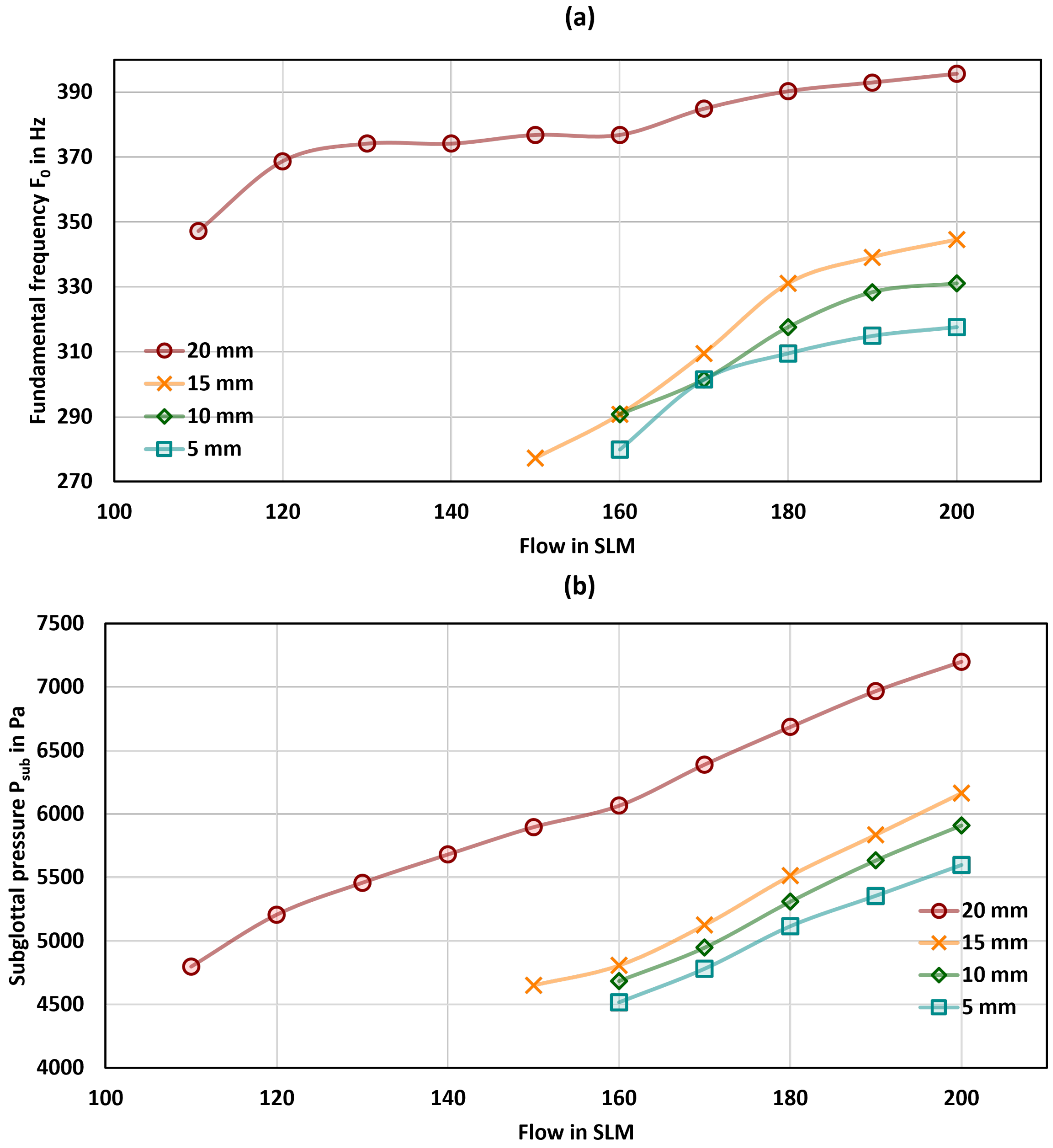

Figure S3. The figure shows the influence of different fiber tension levels for the MLM4 model. Illustrates (**a**) the F_0 in Hz and (**b**) the P_{sub} in Pa as a function of flow in SLM.

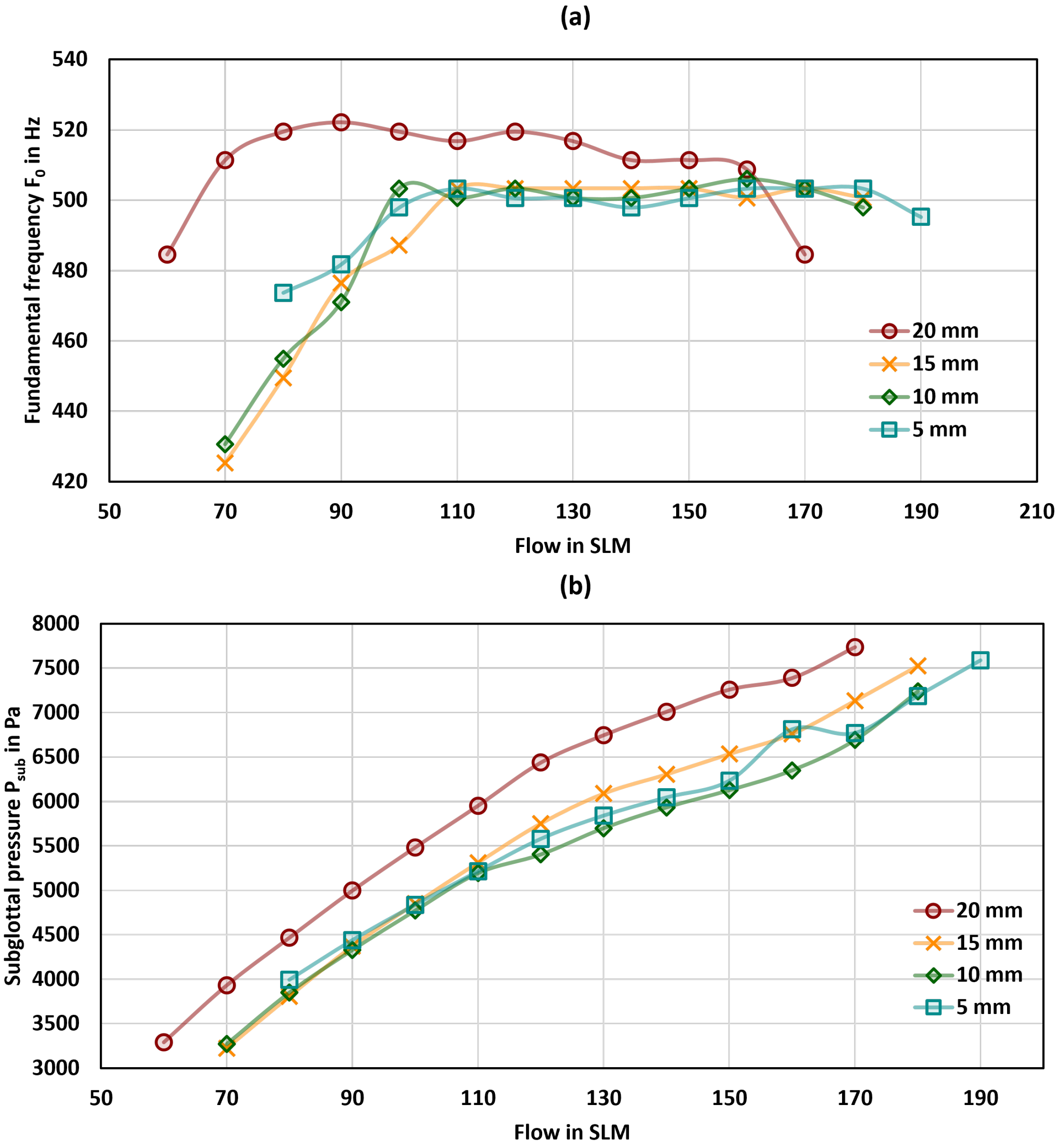

Figure S4. The figure shows the influence of different fiber tension levels for the MLM5 model. Illustrates (**a**) the F_0 in Hz and (**b**) the P_{sub} in Pa as a function of flow in SLM.

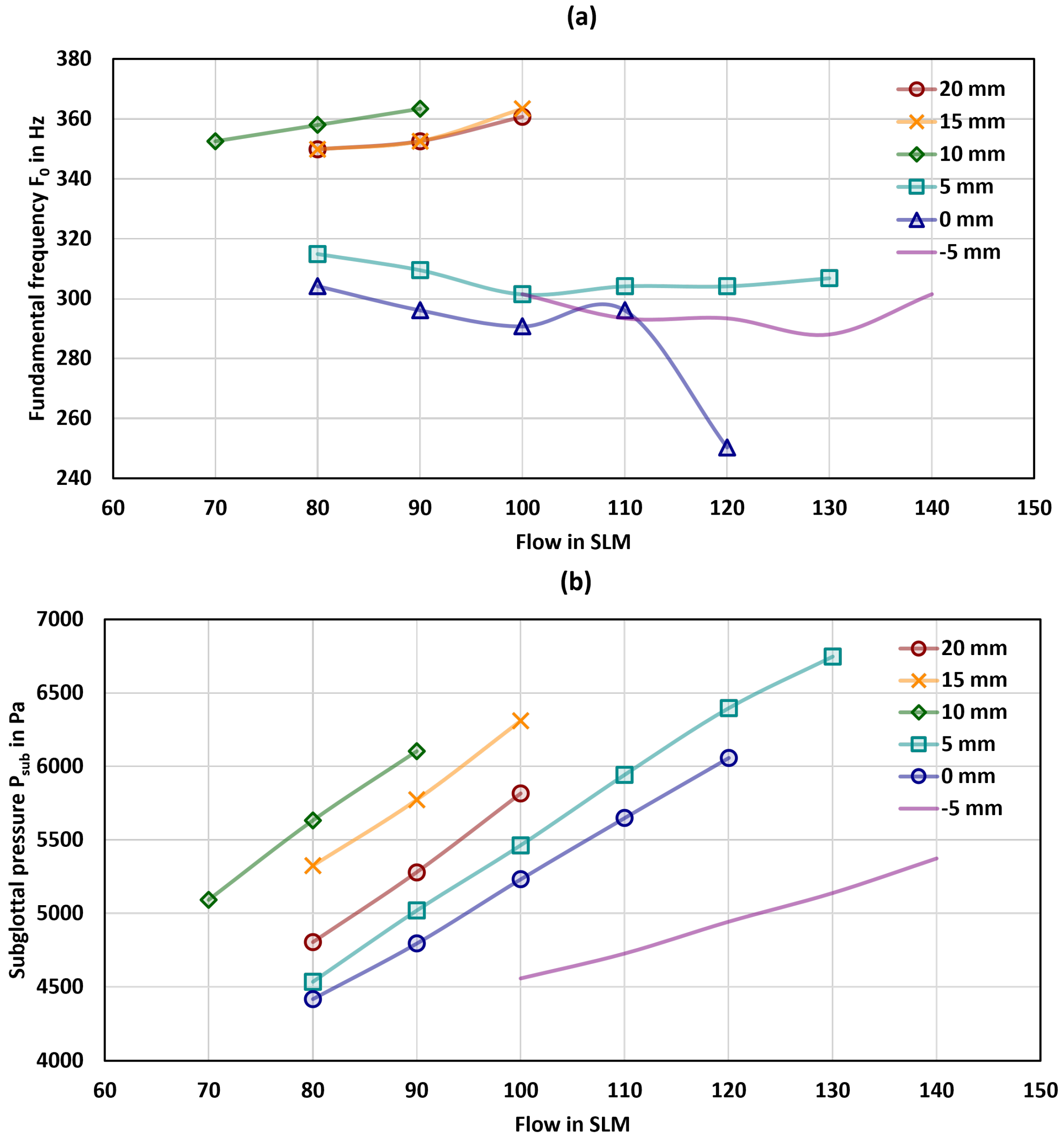

Figure S5. The figure shows the influence of different fiber tension levels for the MLM6 model. Illustrates (**a**) the F_0 in Hz and (**b**) the P_{sub} in Pa as a function of flow in SLM.
